# Effect of Virtual Reality Exercises on the Cognitive Status and Dual Motor Task Performance of the Aging Population

**DOI:** 10.3390/ijerph18158005

**Published:** 2021-07-28

**Authors:** Hadi Nobari, Saeed Rezaei, Mahmoud Sheikh, Juan Pedro Fuentes-García, Jorge Pérez-Gómez

**Affiliations:** 1 Department of Physical Education and Sports, University of Granada, 18010 Granada, Spain; 2 HEME Research Group, Faculty of Sport Sciences, University of Extremadura, 10003 Cáceres, Spain; jorgepg100@gmail.com; 3 Departments of Physical Education and Sport Sciences, University of Tehran, Tehran 6619-14155, Iran; saeed_rezaye@yahoo.com; 4 Didactic and Behavioral Analysis of Sports (ADICODE) Research Group, Faculty of Sport Sciences, University of Extremadura, 10003 Cáceres, Spain; jpfuent@unex.es

**Keywords:** virtual driving, physical activity, behavioral status, mental state, older men

## Abstract

Aging is a global phenomenon affecting numerous developed and developing countries. During this process, the functional state of the body, especially the cognitive state, declines. This research investigated the impact of virtual reality exercises on the cognitive status and dual-task performance in the elderly of Tabriz city, Iran. Forty men with a mean age of 71.5 were selected and assigned to either the experimental (*n* = 20) or control groups (*n* = 20). Both groups completed the Mini-Mental State Examination for cognitive status. The pre-test was performed through the Timed Up and Go test (TUG) along with a countdown of numbers. Then, the experimental group practiced virtual driving for six weeks, while the control group received no treatment. After the treatment, both groups completed the post-test. At each stage, the test was performed as a dual motor task as well. Data were analyzed using the paired *t*-test and the independent sample *t*-test to show the intra-group and inter-group differences, respectively. The results showed a significant improvement in the cognitive status and dual-task performance of the elderly men after the six-week training period, which was also significant compared to the control group. Virtual reality driving can be used to improve the cognitive status and dual task performance of elderly men.

## 1. Introduction

Aging is a global phenomenon and represents the improvement of public health. Recent studies have shown that the elderly population has increased dramatically in recent years [1]. According to the World Health Organization, the number of elderly people will exceed 780 million in 2025 [2]. However, this phenomenon is also marked by profound inequalities, such as those revealed by indicators such as life expectancy at birth. For example, while Japan has the highest life expectancy rate of 82.2 years, life expectancy in several African countries is almost half of that. Health costs increase with age [3], especially in the last two years of life, regardless of age [4]. Since people now live longer than ever before, ensuring the quality health of their remaining years is crucial. 

Brucellosis in the aging population causes fundamental changes in different body systems. Morphological and biochemical changes in different parts of the brain, including sighing and frontal cortex, lead to decreased cognitive features [1,5], additionally, it leads to changes in the musculoskeletal system. In this sense, different studies have shown that physical exercise positively influences different brain functions, for example, attention [6], having carried out studies to discover the effects of aerobic physical exercise on brain neurophysiological activity during the resolution of a selective attention test [7] or working memory performance [8]. Thus, there is evidence that participation in physical activity may modify white matter integrity and the activation of regions key to cognitive processes, although additional larger hypothesis-driven studies are needed to replicate findings [9]. All of the above demonstrates that physical activity can have a positive effect on memory, executive functions, and the anti-aging resilience of the brain, by influencing certain genes associated with neuroprotective signaling [10].

The increase in spontaneous body fluctuations [11] and the high prevalence of falling down in the elderly [12] are signs of degenerative changes in the elderly. All of these changes can reduce the efficiency of dual-task performance in the elderly. In general, it can be argued that when a person grows older, the ability to simultaneously do things decreases [13]. This was demonstrated in a study of the neurophysiological differences between women with fibromyalgia (FM) and healthy controls during a dual-task, which showed women with FM had the same brain activity pattern during single-task and dual-task conditions, whereas healthy controls seemed to adapt their brain activity to the different task commitment [14]. Performing most daily activities such as dressing, brushing, and shopping, when it comes to processing external information, requires careful control and coordination in balance. Several studies have shown disorders in controlling physical force in the elderly, associated with decreased information processing when performing cognitive and motor tasks [15,16]. In recent years, a team of researchers has used a new method to evaluate balance control using the dual-task method. In this method, people are asked to perform two postural and cognitive or postural and motor tasks simultaneously [17]. The dual-task method is a useful method, as our daily activities in the standing position are dual tasks. In addition, everyday activities that require standing rarely occur individually [18]. Driving is a complex skill with the safe operation of a motor vehicle requiring good vision, high motor function, and high cognitive function. Some older drivers have difficulties when they drive in unfamiliar areas, according to an investigation carried out with five hundred and thirty-four drivers, aged ≥ 65 years, who completed a mail-out survey, in which 59.5% considered their abilities as poor or fair rather than good, with the most common strategies regularly used by older drivers to find their way being the use of a street directory whilst driving (61.9%) and pulling over to check a map (55.1%) [19].

Created by computer graphics and 3D screens, virtual reality (VR) offers potential solutions to the challenges posed by traditional research [20]. Recent innovations have made the technology portable, inexpensive, easily accessible, and have deleted the need for an all-inclusive driving simulator and motion tracking tools. This technology is perfectly safe to simulate and practice dangerous skills because the virtual environment is composed of graphics and users are not exposed to physical risks. Researchers have used VR as an education, practice, and rehabilitation tool [21]. Unlike the real world, the virtual world can be tailored to each individual’s capacities and needs, and thus, it provides great flexibility of virtual experiences and tasks, including sequences of functional movements necessary to achieve autonomy in real-life activities. Analyzing and controlling all virtual elements of activity by the occupational therapist lead to virtual achievements that cannot be achieved in real life due to disability or environmental limitations. These achievements increase motivation, commitment, and adherence to the rehabilitation process [22]. Herrero et al. (2015) also investigated the impact of VR on the reaction time of children with autism. Twenty-seven children, 12.6 years of age on average, with high functioning autism participated in the study. The experimental group performed 15 sessions of VR training, 20 min per session. The results showed that VR exercises significantly reduced the reaction time of these children [23]. A very important feature in the technique of VR that was also considered in the present study was the presentation of activity, which is a completely activity-based approach. In this sense, the aim of this study was to analyze the effects of a 24-week exergame intervention and 24 weeks of detraining on lower-limb strength, agility, and cardiorespiratory fitness in women with FM. After the detraining period, lower-limb strength and agility returned to their baseline level, but improvements in cardiorespiratory fitness were sustained over time, so it may have changed the lifestyle of these women, which could explain why cardiorespiratory fitness improvements remained after the detraining period [24]. This attitude is the reverse of the attitude in the pattern of behavior circuit activity, in which activities are divided into their components and each component is carried out separately until success is achieved [25]. In this study, according to the activity-based attitudes of activities, activities were presented to the elderly as a whole, and the person was busy solving in-game problems until success was achieved. In a virtual environment, all characteristics of activity such as duration, intensity, and type of feedback can be changed based on the purpose of treatment and ability of the individual [26]. People can also see their motor results and correct them if necessary [27]. With regard to cognitive status in the older ages, it can be said that aging is associated with significant changes in memory, intelligence, perception, metacognition, recalling, problem solving, and other cognitive abilities, and there is conclusive evidence of the reduction of the processing and function of memory in aging. 

Liao et al., (2019) conducted a 12-week VR-based physical and cognitive training program which led to significant improvements in walking performance and dual-task performance that may be due to improved executive performance [26]. Wu et al., (2020), who reviewed 15 articles on the effects of VR exercises on the cognitive status of patients suffering from cognitive impairment, concluded that VR exercises are useful as a treatment method for people with cognitive impairments [28]. In 2014, Miller et al. conducted a systematic review on the effectiveness of VR and play systems at home in order to improve areas related to the health of the elderly. The evidence derived from 14 studies proved the effectiveness of VR and play systems by the elderly at home, which helped overcome disorders, motor limitations, and poor participation due to their bias in relation to high risks [29]. Davoodeh investigated the effect of VR games on the reaction time of elderly men, in two 15-person control and experimental groups [30]. The experimental group underwent eight weeks (three 45-min sessions a week) of VR exercise, which improved the reaction time of the elderly. VR exercises are therefore shown to be one of the safe and non-invasive methods in the literature for rehabilitation of the elderly [30]. Taking the limitations that elderly people face into consideration, one of the most attractive and non-invasive methods is the VR approach, but the study of various tools of VR and its effects on various physical and psychological factors is still in its infancy. The aim of the present study was to investigate the effect of VR exercise on the cognitive status and dual cognitive task performance in elderly men. 

## 2. Materials and Methods

### 2.1. Participants

The present study is a quasi-experimental study that was conducted in a pre-test and post-test design. The statistical population consisted of all of the elderly men in the Tabriz municipality sports organization (average age of 71.5). First, a list was purposefully prepared among the elderly people of this organization, based on the inclusion criteria of the study, then, 40 eligible individuals were randomly selected, and ultimately, randomly divided into control groups with specifications (71.7 ± 2.4 yrs, confidence interval (CI) 95%, 70.6 to 72.7; 69.3 ± 7.9 kg, CI 95%, 65.9 to 72.8) and experimental groups with specifications (71.4 ± 2.64 yrs, CI 95%, 70.2 to 72.6; 68.8 ± 7.3 kg, CI 95%, 65.6 to 72.0). At first, all of the elderly men completed the personal information form. The selected persons, based on this form, should meet the following criteria for being included as a sample: (a) physical and mental health and the ability to perform low-intensity physical activities, (b) without a record of surgery and special illness, and (c) having a driving license and driving experience. Informed consent was given by all of the patients when collecting their clinical data. The study was approved by the Sport Science Research Institute Ethics Committee (IR.SSRI.REC.1399.851). 

### 2.2. Study Design

First, a list with the names of all of the elderly members of the Tabriz municipality sports organization was prepared, and then a score or number was allocated to each of them, and finally, the required number of men was selected using a random numbers table. Among the eligible ones, 40 elderly men with an average age of 71.5 years were chosen and randomly divided into two groups: the control group or the experimental group (each group had 20 men). The control group took the pre-test and post-test with the cognitive status questionnaire and the Timed Up and Go test (TUG) at the same time as with the countdown of numbers, and performed their daily activities during the treatment period. In addition to participating in the pre-test and post-test, the experimental group participated in the six-week exercise protocol.

All participants completed the cognitive status questionnaire for the pre- and post-test, and the dual-task of sitting and standing with a countdown of numbers was used. The control group went about their daily routines, while the experimental group practiced driving for six weeks with the exercise protocol consisting of three sessions for 20 min per week. 

### 2.3. Timed Up and Go Test (TUG)

The Mini-Mental State Examination [31], which is a practical method for grading cognitive patients, was used and tested five aspects of cognitive function: orientation, recording, attention, calculation, and recall. The maximum score is 30, and the scores 23 and below shows disorder (0–17 shows severe cognitive disorder, 18–23 shows mild to moderate disorder, and 24–30 shows no disorder). The mental state of all of the participants was assessed by a specialist clinician. 

Dual-task assessment was performed using the Timed Up and Go test (TUG), as a quick method to determine the balance problems affecting the motor skills of the elderly person’s daily life. The TUG test consists of three stages: (i) standing up from the chair, (ii) walking, (iii) turning and coming back. Participants were expected to perform this test in the least possible time. They were asked to perform the TUG test under different conditions [32]. The TUG test was performed and the TUG test was also carried out along with a cognitive task (dual cognitive task). The dual cognitive task and the TUG test were performed simultaneously with the countdown of 15 random numbers, in which the participants were evaluated using the pre-test. The experimental group participated in the six-week treatment (3 × 40-min sessions a week), and played the driving game using a computer equipped with a steering wheel and pedals. The steering wheel and pedals with specifications (Ferrari Challenge Racing Wheel) were utilized in conjunction with an ASUS laptop (G551JW) (ASUS, Taipei, Taiwan). Each participant received between 10 and 15 min extra time in the first session to provide the necessary training on how the gadget worked and to become familiar with the game.

### 2.4. Statically Analysis

To investigate the normality of data distribution and variance analysis, Shapiro–Wilk and Leven’s tests were used. Due to the normality of data distribution and variance analysis, a paired t-test was used to investigate intra-group differences, and an independent sample t-test was used to investigate inter-group differences. Data were analyzed by the Statistical Package for the Social Sciences (SPSS) software, version 21 (IBM, San Diego, CA, USA), at a significance level of ≤0.05. 

## 3. Results

As Table 1 shows, there was no significant difference between the pre-test and post-test scores in the control group, but there was a statistically significant difference between the two test results in the experimental group. The results of the paired *t*-test indicated that in the experimental group, there was a significant increase in the post-test scores in cognitive status (t = 4.72, *p* = 0.04) and a significant decrease in the dual-task performance (t = −3.49, *p* = 0.02).

As Table 1 shows, the scores of cognitive status had a significant difference in the experimental group compared to the control group (*p* = 0.013), in addition, there was a significant difference in the scores of the dual-task performance in the experimental group compared to the control group (*p* = 0.020).

## 4. Discussion

In this study, we investigated the effect of VR exercise on the cognitive status and dual cognitive task performance in elderly men in Tabriz. The results showed that VR exercises significantly influenced the cognitive status and dual task performance in the elderly. 

There has recently been a lot of interest in using VR environments in the treatment of various psychological disorders, such as those related to FM [33]. Alternatively, creating various treatment environments with this method has motivated the patients to take a more active role in their own treatment. Since it creates a safe environment and conditions for facing different situations, patients and health care professionals have been more motivated to use VR in treatment. The decrease in sensory and motor integration is part of the aging process, which leads to elderly people facing numerous challenges while coordinating their eyes and hands, eyes and feet, and the two hands, if necessary, for many daily activities. Kinect-based VR exercise is a new technology that is increasingly used in rehabilitation for different people [34]. Findings in our study showed that the VR exercise group (i.e., the experimental group) showed a significant decrease in the cognitive status and dual-task performance compared in the intra-group (i.e., the pre-test and post-test) and inter-group in the post-test stage compared to the control group [35]. A systematic review demonstrated that video game-based interventions help promote physical health (i.e., balance, mobility, strength, physical fitness, and walking performance/gait parameters) and mental health (i.e., balance confidence, executive functions, reaction time, and processing speed) among older adults. It can also be used by researchers in this field to inform their design decisions. We have listed guidelines that can be used to frame future research in the area and enhance its quality [36].

Driving is a complex skill that involves the safe operation of a motor vehicle that requires good sight and high motor and cognitive function [37]. Driving practice as VR engages cognitive status and dual task performance ability to the maximum. Hollman et al. stated that elderly people have a slower walking speed than middle-aged and young people, and at the time of performing a dual-task, this speed decreases more. Additionally, the changes in walking throughout normal and dual task performance occur more in the elderly [38]. This issue is especially important since transport can still be an issue in later life due to physiological and cognitive challenges, with older people being generally skeptical of potential future transport, although they are welcoming of the technologies that reduce physical difficulty in mobility, and provide real-time information [39].

Another finding of our study showed VR exercises have a significant effect on the cognitive status of the elderly, and it is in line with the results of the research studies conducted by Coyle et al. [40], Roberts [41], Chandler [42], Baker [43], and Lin [44]. The results of our study showed positive effects for VR intervention on the main dependent variables, cognitive and physical performance. These results are interesting, considering that common daily life activities are rarely carried out as a single-task but rather require the ability to perform two or more tasks simultaneously [45]. The studies show that there is a decrease in the efficacy of at least one of the tasks simultaneously performed, as a consequence of competition for attentional resources in dual-task conditions [46], resources that decrease when age is advanced [47]. The effect of VR interventions in the cognition group is consistent with the results of a systematic review by Coyle et al. [40]. Coyle et al. showed that VR improved cognitive function intervention of participants with cognitive impairment, on average. A recent VR study reported that executive functions were improved after VR intervention.

Barry investigated the effects of sports games using Xbox Kinect versus traditional exercises without virtual stimuli in gyms on postural control, technology acceptance, psychological factors, and exercise intensity in healthy adults [48]. The study, conducted on 50 healthy active adults, with an average age of 33.8 years, showed that after four weeks of exercise heart rate was equal in both groups, the Borg pressure perception scale was significantly lower in the Kinect group, and that overall, the results showed that postural control, technology acceptance, and psychological factors were improved using Kinect. Improvement of cognitive status and dual-task performance in the VR group can be attributed to the positive ability of this method to integrate the positive benefits of therapeutic techniques of repeated exercises of motion observation, motion imagination, and motor imitation, which causes the plasticity of the nervous system through mirror neurons [48].

Structural interference occurs when the physical or neural structures of the source decrease, which often occur in the implementation of two simultaneous motor tasks. In the interpretation of the present study, by performing two simultaneous motor tasks, the different senses involved in the implementation of tasks (e.g., proprioception and vision) have more practice, and also during the implementation of these tasks by moving the hands, it is likely that the change of location of the center of gravity has occurred, which has somehow led the person to use a more effective strategy to maintain balance, leading to greater balance [5]. In addition, with the continuous exercise of dual motor task ability, structural interference between the two tasks can also be reduced. Overall, it can be argued that dual-task training leads to the simultaneous involvement of postural control processes and balance with cognitive processes. It is interesting here to consider the task prioritization competition model, where older adults prioritize balance and postural stability over cognitive performance in a dual-task with dictions [49].

One of the limitations of this study was the small statistical sample. Another suggestion is to investigate this method on different genders and compare them in terms of the effect of the cognitive status and dual task performance. We hope this can pave the way for the acceptance and wide use of this method by healthcare professions in the future. 

## 5. Conclusions

The results of this quasi-experimental study showed the positive benefits of the VR intervention method in improving the cognitive status and dual-task performance in the elderly population, and as a result, this treatment method can be used to improve some of the challenges our aging population is facing around the world. Given the interesting results of the present study and the effectiveness of VR exercises for the measured variables, we strongly recommend researchers conduct this study design in the future with a larger statistical population as well as between men and women separately. 

## Figures and Tables

**Table 1:** Results of paired and independent *t*-test for cognitive tests and dual-task performance.

Assessments	Groups	Pre-Intervention	Post-Intervention	Within Groups		Between Groups			
				t	*p*	t	*p*	t	*p*
Cognitive status	Control	22.07 ± 2.22	22.47 ± 2.10	0.67	0.162	−0.30	0.767	−2.67	0.013 ^$^
	Experimental	22.33 ± 2.64	24.67 ± 2.41	4.72	0.04 *				
Dual-task	Control	14.40 ± 2.17	14.27 ± 2.28	−0.97	0.124	0.37	0.714	2.46	0.020 ^$^
	Experimental	14.13 ± 1.77	12.40 ± 1.84	−3.49	0.021 *				

* represent significant difference within groups after intervention, at a significance level of ≤0.05. ^$^ represent significant difference between groups after intervention, at a significance level of ≤0.05.

## Data Availability

The datasets used and/or analyzed during the current study are available from the corresponding author upon reasonable request.

## References

[1] Amente T., Kebede B. (2016). Determinants of health service utilization among older adults in Bedele Town, illubabor zone, Ethiopia. J. Diabetes Metab..

[2] Maarsingh O.R., Dros J., Schellevis F.G., van Weert H.C., Bindels P.J., van der Horst H.E. (2010). Dizziness reported by elderly patients in family practice: Prevalence, incidence, and clinical characteristics. BMC Fam. Pract..

[3] WHO (2014). Ageing and Life Course. http://www.who.int/ageing/en.

[4] Statistical Center of Iran (2020). General Population and Housing Census. http://www.amar.org.ir/Default.aspx?tabid=133.

[5] Siu K.-C., Woollacott M.H. (2007). Attentional demands of postural control: The ability to selectively allocate information-processing resources. Gait Posture.

[6] De Bruin E.I., van der Zwan J.E., Bögels S.M. (2016). A RCT comparing daily mindfulness meditations, biofeedback exercises, and daily physical exercise on attention control, executive functioning, mindful awareness, self-compassion, and worrying in stressed young adults. Mindfulness.

[7] Ferro E., Cid F., Muñoz H., Aburto B., Nogales O., Pérez A. (2019). Effects of a session of physical exercise on the neurophysiological activity during the resolution of a test of selective attention [Efectos de una sesión de ejercicio físico sobre la actividad neurofisiológica durante la resolución de una prueba de atención selectiva]. FEADEF.

[8] Hsieh S.-S., Fung D., Tsai H., Chang Y.-K., Huang C.-J., Hung T.-M. (2018). Differences in working memory as a function of physical activity in children. Neuropsychology.

[9] Valkenborghs S.R., Noetel M., Hillman C.H., Nilsson M., Smith J.J., Ortega F.B., Lubans D.R. (2019). The impact of physical activity on brain structure and function in youth: A systematic review. Pediatrics.

[10] Corpas R., Solana E., De la Rosa A., Sarroca S., Griñán-Ferré C., Oriol M., Corbella E., Rodríguez-Farré E., Vina J., Pallàs M. (2019). Peripheral maintenance of the axis SIRT1-SIRT3 at youth level may contribute to brain resilience in middle-aged amateur rugby players. Front. Aging Neurosci..

[11] Siu K.-C., Chou L.-S., Mayr U., van Donkelaar P., Woollacott M.H. (2009). Attentional mechanisms contributing to balance constraints during gait: The effects of balance impairments. Brain Res..

[12] Shumway-Cook A., Woollacott M.H. (2007). Motor Control: Translating Research into Clinical Practice.

[13] Shumway-Cook A., Brauer S., Woollacott M. (2000). Predicting the probability for falls in community-dwelling older adults using the Timed Up & Go Test. Phys. Ther..

[14] Villafaina S., Fuentes-García J.P., Cano-Plasencia R., Gusi N. (2020). Neurophysiological Differences Between Women With Fibromyalgia and Healthy Controls During Dual Task: A Pilot Study. Front. Psychol..

[15] Lundin-Olsson L., Nyberg L., Gustafson Y. (1998). Attention, frailty, and falls: The effect of a manual task on basic mobility. J. Am. Geriatr. Soc..

[16] Sandhu J.S., Paul M., Agnihotri H. (2007). Biofeedback approach in the treatment of generalized anxiety disorder. Iran. J. Psychiatry.

[17] Saboor M., Kamrani A., Momtaz Y.A., Sahaf R. (2019). Prevalence and associated factors of potentially inappropriate medications among Iranian older adults. Med. Glas..

[18] Mishra N. (2015). Comparison of effects of motor imagery, cognitive and motor dual task training methods on gait and balance of stroke survivors. Indian J. Occup. Ther..

[19] Bryden K.J., Charlton J.L., Oxley J.A., Lowndes G.J. (2013). Self-reported wayfinding ability of older drivers. Accid. Anal. Prev..

[20] Neri S.G., Cardoso J.R., Cruz L., Lima R.M., De Oliveira R.J., Iversen M.D., Carregaro R.L. (2017). Do virtual reality games improve mobility skills and balance measurements in community-dwelling older adults? Systematic review and meta-analysis. Clin. Rehabil..

[21] Desapriya E., Harjee R., Brubacher J., Chan H., Hewapathirane D.S., Subzwari S., Pike I. (2014). Vision screening of older drivers for preventing road traffic injuries and fatalities. Cochrane Database Syst. Rev..

[22] Chen K.B., Xu X., Lin J.-H., Radwin R.G. (2015). Evaluation of older driver head functional range of motion using portable immersive virtual reality. Exp. Gerontol..

[23] Herrero D., Crocetta T., Massetti T., de Moraes I., Trevizan I., Guarnieri R. (2015). Total reaction time performance of individuals with autism after a virtual reality task. Int. J. Neurorehabilit..

[24] Villafaina S., Borrega-Mouquinho Y., Fuentes-García J.P., Collado-Mateo D., Gusi N. (2020). Effect of exergame training and detraining on lower-body strength, agility, and cardiorespiratory fitness in women with fibromyalgia: Single-blinded randomized controlled trial. Int. J. Environ. Res. Public Health.

[25] Shin S.-S., An D.-H. (2014). The effect of motor dual-task balance training on balance and gait of elderly women. J. Phys. Ther. Sci..

[26] Liao Y.-Y., Chen I., Lin Y.-J., Chen Y., Hsu W.-C. (2019). Effects of virtual reality-based physical and cognitive training on executive function and dual-task gait performance in older adults with mild cognitive impairment: A randomized control trial. Front. Aging Neurosci..

[27] Enoka R.M., Fuglevand A.J. (2001). Motor unit physiology: Some unresolved issues. Muscle Nerve Off. J. Am. Assoc. Electrodiagn. Med..

[28] Wu J., Ma Y., Ren Z. (2020). Rehabilitative Effects of Virtual Reality Technology for Mild Cognitive Impairment: A Systematic Review With Meta-Analysis. Front. Psychol..

[29] Miller K.J., Adair B.S., Pearce A.J., Said C.M., Ozanne E., Morris M.M. (2014). Effectiveness and feasibility of virtual reality and gaming system use at home by older adults for enabling physical activity to improve health-related domains: A systematic review. Age Ageing.

[30] Davoodeh S., Sheikh M., Homanian Sharif Abadi D., Bagherzadeh F. (2020). The effect of virtual reality games on the mental health of the girls with normal body mass index and above 30. J. Psychol..

[31] Vertesi A., Lever J.A., Molloy D.W., Sanderson B., Tuttle I., Pokoradi L., Principi E. (2001). Standardized Mini-Mental State Examination. Use and interpretation. Can. Fam. Physician.

[32] Almajid R., Tucker C., Wright W.G., Vasudevan E., Keshner E. (2020). Visual dependence affects the motor behavior of older adults during the Timed Up and Go (TUG) test. Arch. Gerontol. Geriatr..

[33] Glasgow A., Stone T.M., Kingsley J.D. (2017). Resistance exercise training on disease impact, pain catastrophizing and autonomic modulation in women with fibromyalgia. Int. J. Exerc. Sci..

[34] Perez-Marcos D., Chevalley O., Schmidlin T., Garipelli G., Serino A., Vuadens P., Tadi T., Blanke O., Millán J.D.R. (2017). Increasing upper limb training intensity in chronic stroke using embodied virtual reality: A pilot study. J. Neuroeng. Rehabil..

[35] Howard M.C. (2017). A meta-analysis and systematic literature review of virtual reality rehabilitation programs. Comput. Hum. Behav..

[36] Xu W., Liang H. (2020). -N.; Baghaei, N.; Wu Berberich, B.; Yue, Y. Health benefits of digital videogames for the aging population: A systematic review. Games Health J..

[37] Ratnasabapathy Y., Chi-Lun Lee A., Feigin V., Anderson C. (2003). Blood pressure lowering interventions for preventing dementia in patients with cerebrovascular disease (Protocol). Cochrane Database Syst. Rev..

[38] Hollman J.H., Childs K.B., McNeil M.L., Mueller A.C., Quilter C.M., Youdas J.W. (2010). Number of strides required for reliable measurements of pace, rhythm and variability parameters of gait during normal and dual task walking in older individuals. Gait Posture.

[39] Musselwhite C. (2019). Older people’s mobility, new transport technologies and user-centred innovation. Towards User-Centric Transport in Europe.

[40] Coyle H., Traynor V., Solowij N. (2015). Computerized and virtual reality cognitive training for individuals at high risk of cognitive decline: Systematic review of the literature. Am. J. Geriatr. Psychiatry.

[41] Roberts A.R., De Schutter B., Franks K., Radina M.E. (2019). Older adults’ experiences with audiovisual virtual reality: Perceived usefulness and other factors influencing technology acceptance. Clin. Gerontol..

[42] Chandler M., Lacritz L., Hynan L., Barnard H., Allen G., Deschner M., Weiner M., Cullum C. (2005). A total score for the CERAD neuropsychological battery. Neurology.

[43] Baker S., Waycott J., Pedell S., Hoang T., Ozanne E. Older people and social participation: From touch-screens to virtual realities. Proceedings of the International Symposium on Interactive Technology and Ageing Populations.

[44] Lin C.X., Lee C., Lally D., Coughlin J.F. Impact of virtual reality (VR) experience on older adults’ well-being. Proceedings of the International Conference on Human Aspects of IT for the Aged Population.

[45] Yuan J., Blumen H.M., Verghese J., Holtzer R. (2015). Functional connectivity associated with gait velocity during walking and walking-while-talking in aging: A resting-state fMRI study. Hum. Brain Mapp..

[46] Crockett R.A., Hsu C.L., Best J.R., Liu-Ambrose T. (2017). Resting state default mode network connectivity, dual task performance, gait speed, and postural sway in older adults with mild cognitive impairment. Front. Aging Neurosci..

[47] Kaliman P., Párrizas M., Lalanza J.F., Camins A., Escorihuela R.M., Pallàs M. (2011). Neurophysiological and epigenetic effects of physical exercise on the aging process. Ageing Res. Rev..

[48] Barry G., Van Schaik P., MacSween A., Dixon J., Martin D. (2016). Exergaming (XBOX Kinect™) versus traditional gym-based exercise for postural control, flow and technology acceptance in healthy adults: A randomised controlled trial. BMC Sports Sci. Med. Rehabil..

[49] Lacour M., Bernard-Demanze L., Dumitrescu M. (2008). Posture control, aging, and attention resources: Models and posture-analysis methods. Clin. Neurophysiol..

